# Molecular mechanism of the effect of Gegen Qinlian decoction on COVID-19 comorbid with diabetes mellitus based on network pharmacology and molecular docking: A review

**DOI:** 10.1097/MD.0000000000034683

**Published:** 2023-11-03

**Authors:** Lin-zi Li, Cong Zhou, Pei Wang, Qing-hua Ke, Jie Zhang, Shan-shan Lei, Zhi-qiang Li

**Affiliations:** a Jingmen Central Hospital, Jingmen, China; b AnKang University, School of Medicine, AnKang, China; c Department of Medicine, Zhejiang Academy of Traditional Chinese Medicine, Hangzhou, China.

**Keywords:** COVID-19, diabetes mellitus, Gegen Qinlian decoction, molecular docking, network pharmacology, pharmacological mechanism

## Abstract

To explore the potential mechanism of Gegen Qinlian decoction (GGQL) in the treatment of COVID-19 comorbid with diabetes mellitus (DM) through network pharmacology and molecular docking, and to provide theoretical guidance for clinical transformation research. Traditional Chinese Medicine Systems Pharmacology Database and Analysis Platform was used to screen the active compounds and targets of GGQL, the targets of COVID-19 comorbid with DM were searched based on Genecards database. Protein-protein interaction network was constructed using String data platform for the intersection of compounds and disease targets, the Gene Ontology and Kyoto Encyclopedia of Genes and Genomes analysis of the intersection targets was performed using DAVID database. Cytoscape software was used to construct the “compound target-pathway (C-T-P)” of GGQL in the treatment of COVID-19 comorbid with DM, the molecular docking platform was used to complete the simulated docking of key compounds and targets. We obtained 141 compounds from GGQL, revealed 127 bioactive compounds and 283 potential targets of GGQL. Quercetin, kaempferol and formononetin in GGQL play a role by modulating the targets (including AR, GSK3B, DPP4, F2, and NOS3). GGQL might affect diverse signaling pathways related to the pathogenesis of coronavirus disease – COVID-19, AGE-RAGE signaling pathway in diabetic complications, IL-17 signaling pathway, human cytomegalovirus infection and Th17 cell differentiation. Meanwhile, molecular docking showed that the selected GGQL core active components had strong binding activity with the key targets. This study revealed that GGQL play a role in the treatment of COVID-19 comorbid with DM through multi-component, multi-target and multi-pathway mode of action, which provided good theoretical basis for further verification research.

## 1. Introduction

Coronavirus disease 2019 (COVID-19) is caused by viral infection with SARS-CoV-2 and has spread rapidly around the world, posing an unprecedented threat to global human health. Since December 2019, SARS-CoV-2 has infected more than 600 million people worldwide and caused more than 6.5 million deaths, with both figures increasing every day. SARS-CoV-2 is mainly transmitted through the respiratory tract and close contact, having the characteristics of strong infectious ability and general susceptibility. The main manifestations of SARS-CoV-2 are fever, dry cough, and fatigue in the early stage, and in severe cases, it can rapidly develop into acute respiratory distress syndrome, multiple organ dysfunction syndrome, coagulation dysfunction, and metabolic acidosis.^[1]^ Furthermore, COVID-19 patients with diabetes mellitus (DM) have a higher promotion of severe cases and a remere like to cause serious adverse outcomes.^[2]^ Previous studies have found the prevalence of DM in a COVID-19-critical group to be 22.2%,^[3,4]^ with another study reporting that the related multivariate hazard ratio for mortality was 3.64.^[5]^

Traditional Chinese medicine (TCM) serves as a strong theoretical framework for the prevention and treatment of diseases and has played an important role in the treatment of COVID-19. Gegen Qinlian Decoction (GGQL) derived from the “Treatise on Febrile Diseases” (shanghanlun) by Zhang Zhongjing. The whole prescription is composed of 4 Chinese herbs (*Radix puerariae, Radix scutellariae, Rhizoma coptidis, and Radix glycyrrhizae*) which has the effect of removing the surface and clearing the interior, which are characterized shows the effects of clearing heat, stopping diarrhea, antibacterial and antiviral. At the same time, the efficacy of GGQL is consistent with the pathogenesis of diabetes mellitus type 2 (T2DM) in the early stage, which is “full of internal heat, damp heat, and internal resistance.” In clinical practice, the decoction is often used as a basic prescription for the prevention and treatment of diabetes, for which its curative effect is significant.^[6]^ Modern pharmacological research has shown that GGQL has multiple pharmacological effects, including hypoglycemic,^[7]^ and anti-viral,^[8]^ effects. The decoction has thus been included in the list of Chinese patent medicine preparations recommended by the Treatment Plan of Traditional Chinese Medicine for COVID-19 in the Guangxi Zhuang Autonomous Region (Trial third edition), and has been applied to patients during both the medical observation period and clinical treatment period of COVID-19 in the Hubei Provincial Hospital of Traditional Chinese Medicine. A clinical study investigating the use of GGQL in the treatment of COVID-19 reported that it can prevent the development of severe disease, significantly improve symptoms; such as cough, chest tightness, nausea, and vomiting; significantly shorten the nucleic acid conversion time, and prevent pulmonary inflammation.^[9]^ However, the therapeutic potential, benefits, and mechanism of GGQL in the treatment of COVID-19 comorbid with DM are still unclear, while its main active compounds and mechanism of action have not yet been elucidated by employing a network pharmacological approach.

Based on systems biology theory, network pharmacology is a method used to systematically analyze the complex relationships between drugs and diseases from a high-level perspective. The research strategy that it encompasses conforms to the characteristics of multi-component, multi-target, and multi-pathway treatment, which is fundamental to TCM. The status of Chinese medicine formula-based research based on network pharmacology provides references and novel avenues for the further study of the pharmacological mechanisms underlying the effectiveness of various TCMs.^[10]^ Molecular docking is an *in silico* technique used in drug design and screening that reveals the characteristics and interactions of receptors and their ligands through electrical force analysis, simulating and predicting their binding modes.^[11]^ In this study, network pharmacology and molecular docking simulations were employed to construct the potential active compounds and targets of GGQL against COVID-19 comorbid with DM. The results are expected to indicate the potential mechanism of action of GGQL in the treatment of COVID-19 comorbid with DM and to provide a theoretical basis for its subsequent clinical application.

## 2. Materials and methods

**Figure 1.**
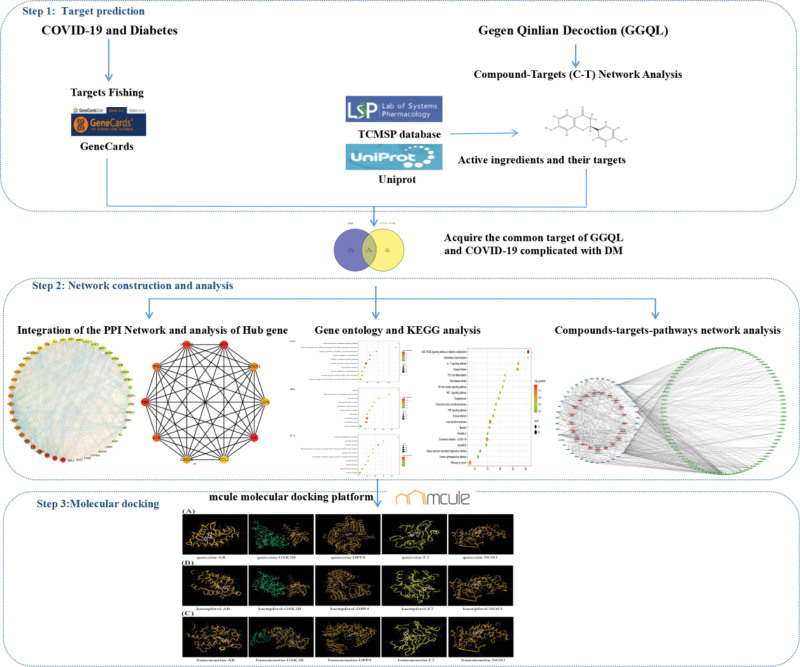
Network pharmacologic research workflow of GGQL in the treatment of COVID-19 complicated with diabetes. GGQL = Gegen Qinlian decoction.

This research adopted network pharmacology and molecular docking to unveil the biochemistry basis and underly mechanisms of GGQL as a treatment for COVID-19 combined with DM. Figure 1 shows an overview of the experimental steps.

### 2.1. Collection and screening of pharmaceutical components and their targets

All candidate herbal compounds of GGQL (*Radix puerariae, Radix scutellariae, Rhizoma coptidis, and Radix glycyrrhizae*) were harvested by using Traditional Chinese Medicine Systems Pharmacology Database and Analysis Platform (TCMSP, http://tcmspw.com/index.php). As the TCMSP suggested, the compounds with oral bioavailability (OB) ≥ 30% show good absorption and slow metabolism property after oral administration. The compounds with drug-likeness (DL) ≥ 0.18 were chemically suitable for drug development. Hence, 2 ADME-related parameters including OB ≥ 30% and DL ≥ 0.18 were employed to identify the potential active compounds in GGQL, the obtained active compounds were adopted as the candidate active compounds.

TCMSP was adopted to screen the targets of candidate active substances in GGQL. The collected targets were confirmed with the Uniprot protein sequence resource (http://www.Uniprot.org/), including name, gene ID, and organism. The active compounds without targets were eliminated and the active compound-target dataset was set up.

### 2.2. COVID-19 combined with DM-related diseases target collection

This study through Genecards database (https://www.genecards.org/) to obtain COVID-19 and DM targets; “COVID-19,” “SARS-CoV-2” and “diabetes mellitus” were used as keywords to collect disease targets, and the intersection targets of the 2 diseases were removed to establish a target database of COVID-19 combined with DM. Target genes were selected with a correlation score ≥ 5 in the GeneCards database. After deleting duplicates, the targets related to COVID-19 combined with DM were obtained.

### 2.3. Protein–protein interaction network construction and Hub gene analysis

The Venn diagram was set up through an online website (https://bioinfogp.cnb.csic.es/tools/venny/index.html) to acquire the common targets for GGQL bioactive compound targets and the COVID-19 combined with DM associated targets.

Then, the common target genes were input into the STRING database (http://string-db.org/) to explore the protein interaction, and the protein–protein interaction (PPI) network was constructed with the Cytoscape. The nodes in PPI network were performed by “Network Analyzer,” and the Hub genes of GGQL against COVID-19 combined with DM were calculated by Cytohubba (http://apps.cytoscape.org/apps/cytohubba) plugin by Matthews correlation coefficient algorithm in this PPI network.

### 2.4. Gene Ontology enrichment and Kyoto Encyclopedia of Genes and Genomes pathway analysis

As an effective bioinformatics tool, the Gene Ontology (GO) analysis can characterize molecular function (MF), cellular components (CC), and biological process (BP) of genes. The Kyoto Encyclopedia of Genes and Genomes (KEGG) enrichment exploration collects databases illustrating biological paths, genomes, drugs and diseases. In this study, we analyzed the GO function and KEGG pathway enrichment of the proteins involved in the PPI network using the DAVID database(https://david.ncifcrf.gov/), and appropriately characterized the pathway associations and GO functions based on their enrichment.

### 2.5. Compound-target-pathway network construction and analysis

We used Cytoscape software to construct the “Compounds-targets-pathways (C-T-P)” network for GGQL as a treatment for COVID-19 combined with DM according to the active compounds in GGQL, intersecting the targeted genes related COVID-19 combined with DM with the GGQL active compounds through the pathway from KEGG analysis. In this network, every compound, target, or pathway is represented by a node and every interaction by an edge. At the same time, the plugin Network Analyzer of Cytoscape 3.7.0 was adopted to analyze.

### 2.6. Molecular docking

The top 3 effective active compounds of GGQL related to COVID-19 combined with DM were docked with the top 5 core targets, respectively, and the binding mode and electrical force of PPI were predicted and obtained. By PDB database (https://www.wwpdb.org/) to download the target protein 3 D structure, from the Pubchem database (https://pubchem.ncbi.nlm.nih.gov/) to download GGQL active ingredient mol2 files, molecular docking was then performed using the molecular docking platform (https://mcule.com). Binding energy was used as an index to evaluate the binding activity and docking effect of the ligand-protein interaction.

## 3. Results

### 3.1. Screening for active compounds and their targets in GGQL

According to TCMSP databases, we obtained 141 active compounds in GCQL (including *Radix puerariae, Radix scutellariae, Rhizoma coptidis*, and *Radix glycyrrhizae*) based on ADME model (OB ≥ 30% and DL ≥ 0.18), active compounds as shown in Table 1.

**Table 1:** Chemical information of active compounds in GGQL.

Herbal medicine	Mol ID	Molecule name	MW	OB (%)	DL
Pueraria lobata	MOL000392	formononetin	268.28	45.97	0.19
	MOL000358	beta-sitosterol	414.79	42.1	0.2
	MOL002959	3’-Methoxydaidzein	284.28	34.97	0.24
	MOL003629	Daidzein-4,7-diglucoside	578.57	39.84	0.71
	MOL012297	puerarin	416.41	56.45	0.39
Coptis chinensis	MOL001454	berberine	336.39	36.86	0.78
	MOL013352	Obacunone	454.56	43.29	0.77
	MOL002894	berberrubine	322.36	35.74	0.73
	MOL002897	epiberberine	336.39	43.09	0.78
	MOL002903	(R)-Canadine	339.42	55.37	0.77
	MOL002904	Berlambine	351.38	36.68	0.82
	MOL002907	Corchoroside A_qt	404.55	104.95	0.78
	MOL000622	Magnograndiolide	266.37	63.71	0.19
	MOL000762	Palmidin A	510.52	35.36	0.65
	MOL000785	palmatine	352.44	64.6	0.65
	MOL000098	quercetin	302.25	46.43	0.28
	MOL001458	coptisine	320.34	30.67	0.86
	MOL002668	Worenine	334.37	45.83	0.87
	MOL008647	Moupinamide	313.38	86.71	0.26
	MOL001689	acacetin	284.28	34.97	0.24
	MOL000173	wogonin	284.28	30.68	0.23
	MOL000228	(2R)-7-hydroxy-5-methoxy-2-phenylchroman-4-one	270.3	55.23	0.2
	MOL002714	baicalein	270.25	33.52	0.21
	MOL002908	5,8,2’-Trihydroxy-7-methoxyflavone	300.28	37.01	0.27
	MOL002909	5,7,2,5-tetrahydroxy-8,6-dimethoxyflavone	376.34	33.82	0.45
	MOL002910	Carthamidin	288.27	41.15	0.24
	MOL002911	2,6,2’,4’-tetrahydroxy-6’-methoxychaleone	302.3	69.04	0.22
	MOL002913	Dihydrobaicalin_qt	272.27	40.04	0.21
	MOL002914	Eriodyctiol (flavanone)	288.27	41.35	0.24
	MOL002915	Salvigenin	328.34	49.07	0.33
	MOL002917	5,2’,6’-Trihydroxy-7,8-dimethoxyflavone	330.31	45.05	0.33
	MOL002925	5,7,2’,6’-Tetrahydroxyflavone	286.25	37.01	0.24
	MOL002926	dihydrooroxylin A	286.3	38.72	0.23
	MOL002927	Skullcapflavone II	374.37	69.51	0.44
	MOL002928	oroxylin a	284.28	41.37	0.23
	MOL002932	Panicolin	314.31	76.26	0.29
	MOL002933	5,7,4’-Trihydroxy-8-methoxyflavone	300.28	36.56	0.27
	MOL002934	NEOBAICALEIN	374.37	104.34	0.44
	MOL002937	DIHYDROOROXYLIN	286.3	66.06	0.23
	MOL000358	beta-sitosterol	414.79	36.91	0.75
	MOL000359	sitosterol	414.79	36.91	0.75
	MOL000525	Norwogonin	270.25	39.4	0.21
Baikal Skullcap	MOL000552	5,2’-Dihydroxy-6,7,8-trimethoxyflavone	344.34	31.71	0.35
	MOL000073	ent-Epicatechin	290.29	48.96	0.24
	MOL000449	Stigmasterol	412.77	43.83	0.76
	MOL001458	coptisine	320.34	30.67	0.86
	MOL001490	bis[(2S)-2-ethylhexyl] benzene-1,2-dicarboxylate	390.62	43.59	0.35
	MOL001506	Supraene	410.8	33.55	0.42
	MOL002879	Diop	390.62	43.59	0.39
	MOL002897	epiberberine	336.39	43.09	0.78
	MOL008206	Moslosooflavone	298.31	44.09	0.25
	MOL010415	11,13-Eicosadienoic acid, methyl ester	322.59	39.28	0.23
	MOL012245	5,7,4’-trihydroxy-6-methoxyflavanone	302.3	36.63	0.27
	MOL012246	5,7,4’-trihydroxy-8-methoxyflavanone	302.3	74.24	0.26
	MOL012266	rivularin	344.34	37.94	0.37
	MOL001484	Inermine	284.28	75.18	0.54
	MOL001792	DFV	256.27	32.76	0.18
	MOL000211	Mairin	456.78	55.38	0.78
	MOL002311	Glycyrol	366.39	90.78	0.67
	MOL000239	Jaranol	314.31	50.83	0.29
	MOL002565	Medicarpin	270.3	49.22	0.34
	MOL000354	isorhamnetin	316.28	49.6	0.31
	MOL000359	sitosterol	414.79	36.91	0.75
	MOL003656	Lupiwighteone	338.38	51.64	0.37
	MOL003896	7-Methoxy-2-methyl isoflavone	266.31	42.56	0.2
	MOL000392	formononetin	268.28	69.67	0.21
	MOL000417	Calycosin	284.28	47.75	0.24
	MOL000422	kaempferol	286.25	41.88	0.24
	MOL004328	naringenin	272.27	59.29	0.21
	MOL004805	(2S)-2-[4-hydroxy-3-(3-methylbut-2-enyl) phenyl]-8,8-dimethyl-2,3-dihydropyrano[2,3-f] chromen-4-one	390.51	31.79	0.72
	MOL004806	euchrenone	406.56	30.29	0.57
	MOL004808	glyasperin B	370.43	65.22	0.44
	MOL004810	glyasperin F	354.38	75.84	0.54
	MOL004811	Glyasperin C	356.45	45.56	0.4
	MOL004814	Isotrifoliol	298.26	31.94	0.42
	MOL004815	(E)-1-(2,4-dihydroxyphenyl)-3-(2,2-dimethylchromen-6-yl) prop-2-en-1-one	322.38	39.62	0.35
	MOL004820	kanzonols W	336.36	50.48	0.52
	MOL004824	(2S)-6-(2,4-dihydroxyphenyl)-2-(2-hydroxypropan-2-yl)-4-methoxy-2,3-dihydrofuro[3,2-g] chromen-7-one	384.41	60.25	0.63
	MOL004827	Semilicoisoflavone B	352.36	48.78	0.55
	MOL004828	Glepidotin A	338.38	44.72	0.35
	MOL004829	Glepidotin B	340.4	64.46	0.34
	MOL004833	Phaseolinisoflavan	324.4	32.01	0.45
	MOL004835	Glypallichalcone	284.33	61.6	0.19
	MOL004838	8-(6-hydroxy-2-benzofuranyl)-2,2-dimethyl-5-chromenol	308.35	58.44	0.38
	MOL004841	Licochalcone B	286.3	76.76	0.19
	MOL004848	licochalcone G	354.43	49.25	0.32
Licorice	MOL004849	3-(2,4-dihydroxyphenyl)-8-(1,1-dimethylprop-2-enyl)-7-hydroxy-5-methoxy-coumarin	368.41	59.62	0.43
	MOL004855	Licoricone	382.44	63.58	0.47
	MOL004856	Gancaonin A	352.41	51.08	0.4
	MOL004857	Gancaonin B	368.41	48.79	0.45
	MOL004860	licorice glycoside E	693.71	32.89	0.27
	MOL004863	3-(3,4-dihydroxyphenyl)-5,7-dihydroxy-8-(3-methylbut-2-enyl) chromone	354.38	66.37	0.41
	MOL004864	5,7-dihydroxy-3-(4-methoxyphenyl)-8-(3-methylbut-2-enyl) chromone	352.41	30.49	0.41
	MOL004866	2-(3,4-dihydroxyphenyl)-5,7-dihydroxy-6-(3-methylbut-2-enyl) chromone	354.38	44.15	0.41
	MOL004879	Glycyrin	382.44	52.61	0.47
	MOL004882	Licocoumarone	340.4	33.21	0.36
	MOL004883	Licoisoflavone	354.38	41.61	0.42
	MOL004884	Licoisoflavone B	352.36	38.93	0.55
	MOL004885	licoisoflavanone	354.38	52.47	0.54
	MOL004891	shinpterocarpin	322.38	80.3	0.73
	MOL004898	(E)-3-[3,4-dihydroxy-5-(3-methylbut-2-enyl) phenyl]-1-(2,4-dihydroxyphenyl)prop-2-en-1-one	340.4	46.27	0.31
	MOL004903	liquiritin	418.43	65.69	0.74
	MOL004904	licopyranocoumarin	384.41	80.36	0.65
	MOL004905	3,22-Dihydroxy-11-oxo-delta (12)-oleanene-27-alpha-methoxycarbonyl-29-oic acid	512.75	34.32	0.55
	MOL004907	Glyzaglabrin	298.26	61.07	0.35
	MOL004908	Glabridin	324.4	53.25	0.47
	MOL004910	Glabranin	324.4	52.9	0.31
	MOL004911	Glabrene	322.38	46.27	0.44
	MOL004912	Glabrone	336.36	52.51	0.5
	MOL004913	1,3-dihydroxy-9-methoxy-6-benzofurano[3,2-c] chromenone	298.26	48.14	0.43
	MOL004914	1,3-dihydroxy-8,9-dimethoxy-6-benzofurano[3,2-c] chromenone	328.29	62.9	0.53
	MOL004915	Eurycarpin A	338.38	43.28	0.37
	MOL004917	glycyroside	562.57	37.25	0.79
	MOL004924	(-)-Medicocarpin	432.46	40.99	0.95
	MOL004935	Sigmoidin-B	356.4	34.88	0.41
	MOL004941	(2R)-7-hydroxy-2-(4-hydroxyphenyl) chroman-4-one	256.27	71.12	0.18
	MOL004945	(2S)-7-hydroxy-2-(4-hydroxyphenyl)-8-(3-methylbut-2-enyl) chroman-4-one	324.4	36.57	0.32
	MOL004948	Isoglycyrol	366.39	44.7	0.84
	MOL004949	Isolicoflavonol	354.38	45.17	0.42
	MOL004957	HMO	268.28	38.37	0.21
	MOL004959	1-Methoxyphaseollidin	354.43	69.98	0.64
	MOL004961	Quercetin der.	330.31	46.45	0.33
	MOL004966	3’-Hydroxy-4’-O-Methylglabridin	354.43	43.71	0.57
	MOL000497	licochalcone a	338.43	40.79	0.29
	MOL004974	3’-Methoxyglabridin	354.43	46.16	0.57
	MOL004978	2-[(3R)-8,8-dimethyl-3,4-dihydro-2H-pyrano[6,5-f] chromen-3-yl]-5-methoxyphenol	338.43	36.21	0.52
	MOL004980	Inflacoumarin A	322.38	39.71	0.33
	MOL004985	icos-5-enoic acid	310.58	30.7	0.2
	MOL004988	Kanzonol F	420.54	32.47	0.89
	MOL004989	6-prenylated eriodictyol	356.4	39.22	0.41
	MOL004990	7,2’,4’-trihydroxy–5-methoxy-3–arylcoumarin	300.28	83.71	0.27
	MOL004991	7-Acetoxy-2-methylisoflavone	294.32	38.92	0.26
	MOL004993	8-prenylated eriodictyol	356.4	53.79	0.4
	MOL004996	gadelaidic acid	310.58	30.7	0.2
	MOL000500	Vestitol	272.32	74.66	0.21
	MOL005000	Gancaonin G	352.41	60.44	0.39
	MOL005001	Gancaonin H	420.49	50.1	0.78
	MOL005003	Licoagrocarpin	338.43	58.81	0.58
	MOL005007	Glyasperins M	368.41	72.67	0.59
	MOL005008	Glycyrrhiza flavonol A	370.38	41.28	0.6
	MOL005012	Licoagroisoflavone	336.36	57.28	0.49
	MOL005013	18α-hydroxyglycyrrhetic acid	486.76	41.16	0.71
	MOL005016	Odoratin	314.31	49.95	0.3
	MOL005017	Phaseol	336.36	78.77	0.58
	MOL005018	Xambioona	388.49	54.85	0.87
	MOL005020	dehydroglyasperins C	340.4	53.82	0.37
	MOL000098	quercetin	302.25	46.43	0.28

There were 141 candidate molecules were selected on basis of coefficients of ADME nature (Per OB ≥ 30%, Per DL ≥ 0.18) from GGQL by TCMSP.

DL = distribution, GGQL = Gegen Qinlian decoction, MW = molecular weight, OB = oral bioavailability, TCMSP = The traditional Chinese medicine systems pharmacology database and analysis platform (http://tcmspw.com/index.php).

The targets of GGQL candidate active compounds were also screened out by TCMSP database. After the active compounds without targets were removed, 127 active compounds and corresponding 283 targets were obtained (see Table S1, Supplemental Digital Content, http://links.lww.com/MD/J457 for details).

### 3.2. COVID-19 combined with DM-related diseases targets

A total of 542 COVID-19 and 1612 diabetes disease-related targets were identified using the Genecards database (correlation score ≥ 5). Based on the results of various databases, a total of 167 targets related to COVID-19 comorbid with DM were finally screened out after reprofiling and taking the intersection targets of the 2 diseases (see Table S2, Supplemental Digital Content, http://links.lww.com/MD/J458 for details).

### 3.3. Prediction of common targets associated with GGQL and COVID-19 comorbid with DM

**Figure 2.**
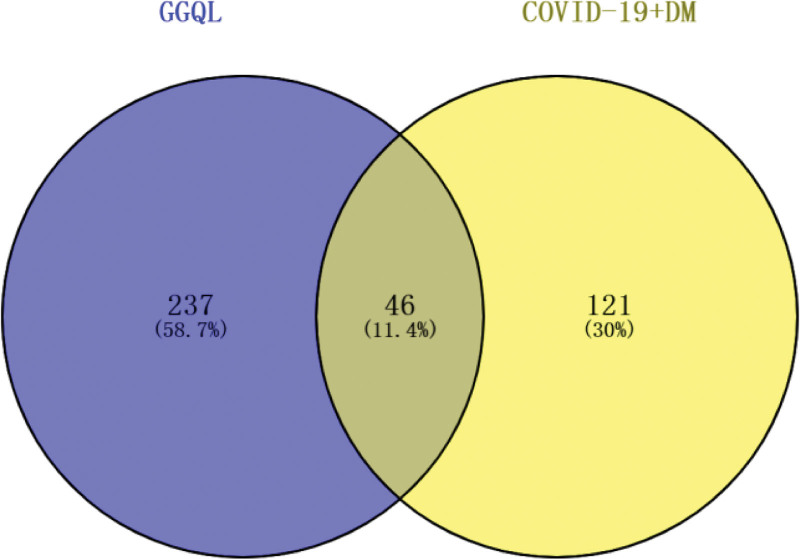
Compound-target network. A Venn diagram was set up by an online website (https://bioinfogp.cnb.csic.es/tools/venny/index.html) to get the 46 common targets of the GGQL bioactive component targets and the COVID-19 complicated with DM associated targets. DM = diabetes mellitus, GGQL = Gegen Qinlian decoction.

A total of 167 targets related to COVID-19 comorbid with DM were obtained through GeneCards database, which were matched with 283 targets of active compounds in GGQL, and 46 targets of active compounds in GGQL acting on COVID-19 comorbid with DM were obtained (see Table 2 for details). Venny2.1.0 (https://bioinfogp.cnb.csic.es/tools/venny/index.html) online mapping tool was used to draw the Venn diagram of drug - disease targets (see Fig. 2). In the figure, blue nodes represent drug targets, yellow nodes represent disease targets, and gray nodes represent the intersection of the 2 targets.

**Table 2:** Target information of the active ingredients in GGQL against COVID-19 complicated with DM.

Disease	Targets number	Targets
COVID-19 complicated with DM	46	AR, DPP4, GSK3B, F2, NOS3, JUN, IL4, BCL2, TGFB1, STAT3, AKT1, VEGFA, TNF, HIF1A, AGTR1, IFNB1, ACE2, MMP3, EGFR, MAPK1, IL10, IL6, TP53, MMP1, STAT1, HMOX1, CYP3A4, CAV1, F3, GJA1, IL1B, CCL2, CXCL8, IL2, SERPINE1, IFNG, ALOX5, NFE2L2, AHR, CRP, CXCL10, SPP1, IGFBP3, FASN, IKBKB, LDLR

AR = androgens receptors, DM = diabetes mellitus, GGQL = Gegen Qinlian decoction.

### 3.4. PPI network and Hub gene analysis

**Figure 3.**
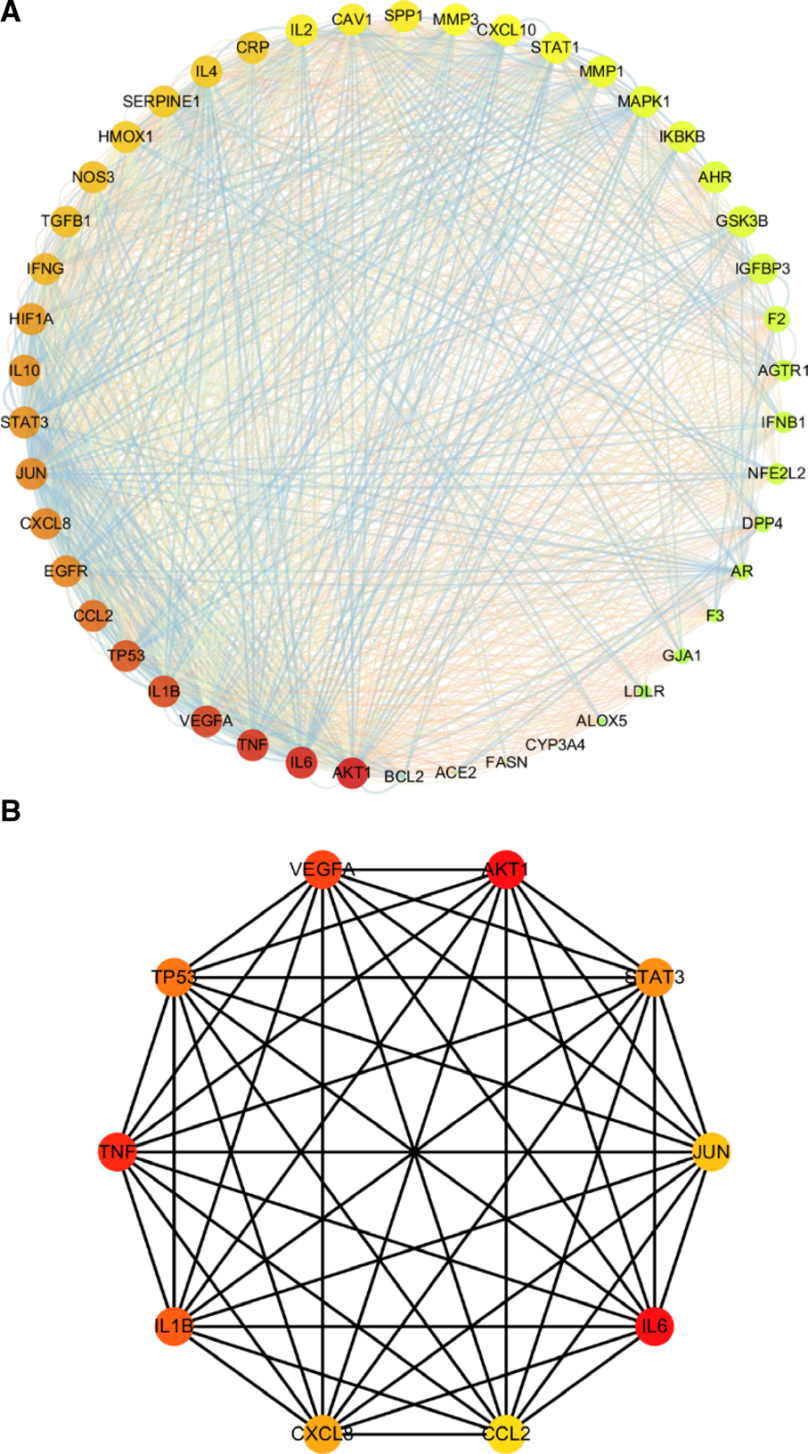
PPI network of GGQL for COVID-19 combined with diabetes targets. (A) These target genes were input into the STRING online website (PPI score > 0.4), PPI network consists of 46 interacting nodes and 599 interacting edges. Nodes refer to core target genes. The sizes of the nodes and edges match the values of the degree and integral markers, respectively. The color of the node represents the degree value. If the color is darker (red), the degree value will be higher. (B) The Hub gene of GGQL against COVID-19 combined with diabetes was calculated by Cytohubba (http://apps.cytoscape.org/apps/cytohubba) plugin by MCC algorithm, the 5 nodes with the largest degree value were chosen as the hub genes, the darker (red) the node color, the higher the score. GGQL = Gegen Qinlian decoction, MCC = Matthews correlation coefficient, PPI = protein–protein interaction.

The obtained 46 common targets of GGQL bioactive compounds and COVID-19 comorbid with DM were input into the STRING website (PPI score > 0.4), and use Cytoscape 3.7.0 software to plot the PPI network (see Fig. 3), which consists of 46 interacting nodes and 599 interacting edges. As shown in Figure 3A, the sizes of nodes and edges correspond to the values of degrees and combined fractions, respectively. The color of the node represents the value of the degree. The darker the color (red), the higher the degree.

Based on Cytoscape plugin Cytohubba, Hub genes were screened in the interaction network. The Matthews correlation coefficient algorithm was used to identify the top 10 Hub genes of GGQL in the treatment of COVID-19 comorbid with DM (see Fig. 3B), which are AKT1, IL6, TNF, VEGFA, IL1B, TP53, CCL2, EGFR, CXCL8 and JUN.

### 3.5. GO enrichment and KEGG pathway analysis

**Figure 4.**
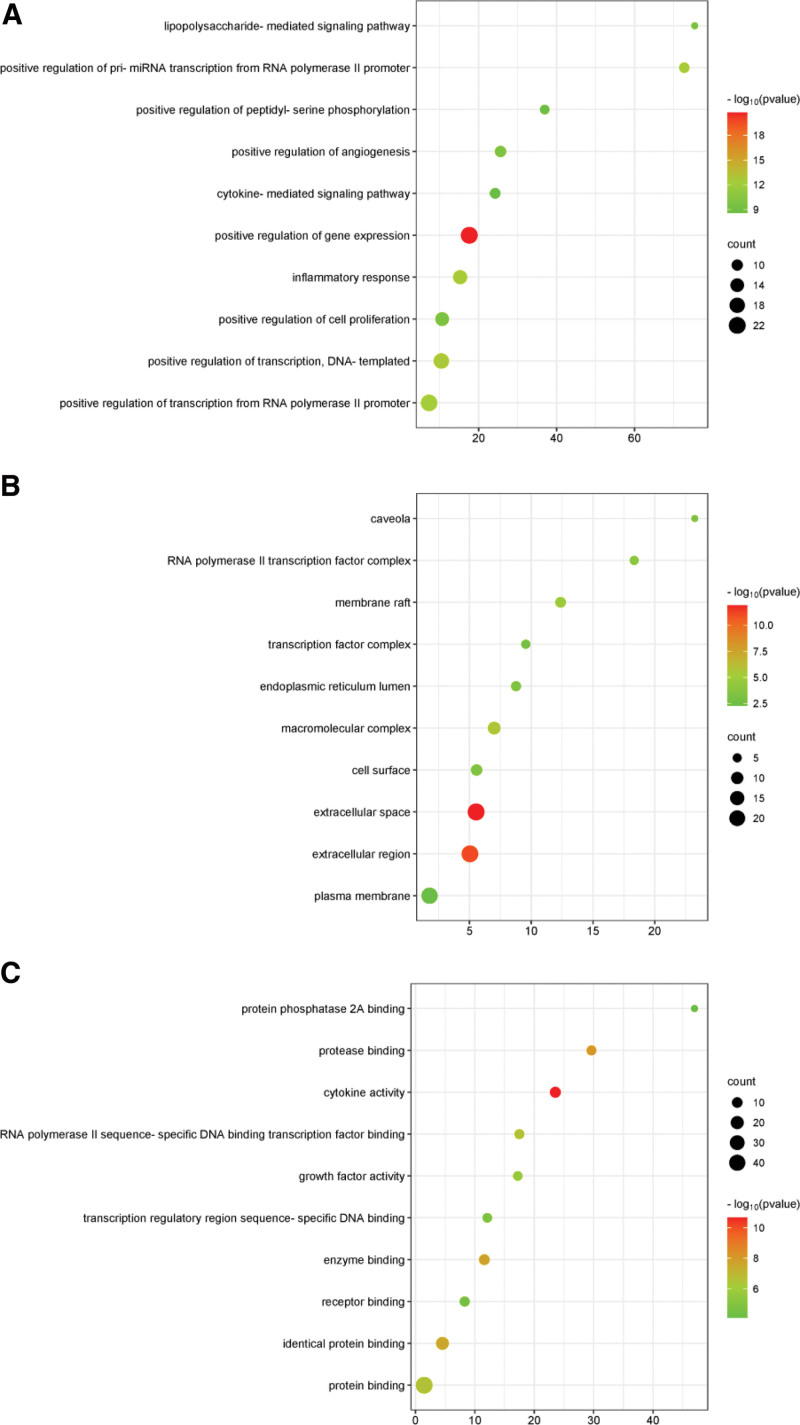
DAVID database was used for GO functional enrichment analysis. (A) Enrichment analysis of BPs, (B) enrichment analysis of MFs, (C) enrichment analysis of CCs. The active compounds of GGQL and the intersection target genes associated with COVID-19 combined with diabetes were imported into the DAVID database for GO analysis. The *Y* axis represents the class of BPs that are significantly enhanced in relation to target genes, the *X* axis represents log10 (*P* value), the size of the dots represents the number of target genes in the pathway, and the color of the dots represents the various FDR ranges. BP = biological process, CC = cellular components, FDR = false discovery rate, GGQL = Gegen Qinlian decoction, GO = Gene Ontology, MF = molecular function.

In order to elucidate the mechanism of GGQL in the treatment of COVID-19 comorbid with DM at the integrated level, GO enrichment analysis was performed on the BPs, MFs and CCs of 46 common targets. Figure 4 lists the top 10 significantly enriched GO items at these targets (false discovery rate < 0.05). The results showed that the targets of GGQL were closely related to 5 BP: positive regulation of gene expression, positive regulation of transcription, DNA-templated, inflammatory response, positive regulation of pri-miRNA transcription from RNA polymerase II promoter, positive regulation of transcription from RNA polymerase II promoter; 5 MFs: cytokine activity, protease binding, enzyme binding, identical protein binding, RNA polymerase II sequence-specific DNA binding transcription factor binding; 5 CCs: extracellular space, extracellular region, macromolecular complex, membrane raft, RNA polymerase II transcription factor complex.

**Figure 5.**
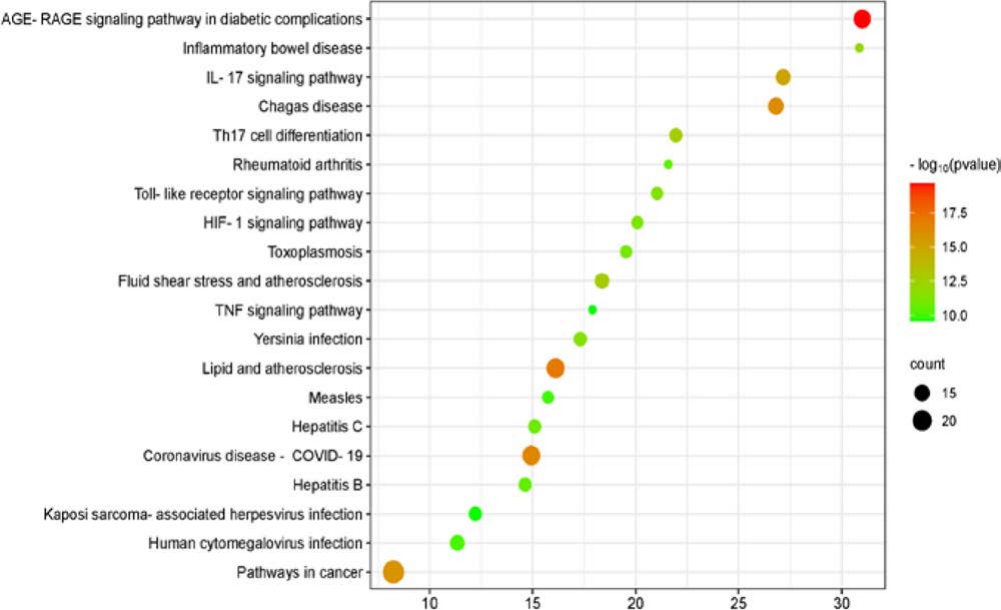
KEGG path analysis using DAVID database. The active compounds of GGQL and the intersection target genes associated with COVID-19 combined with diabetes were imported into the DAVID database for KEGG pathway analysis. The *Y* axis represents the greatly improved BP associated with the target gene, the *X* axis represents log10 (*P* value), the size of the dots represents the number of target genes in the pathway, and the color of the dots represents the different FDR ranges. BP = biological process, FDR = false discovery rate, KEGG = Kyoto Encyclopedia of Genes and Genomes, GGQL = Gegen Qinlian decoction.

As shown in Figure 5, we analyzed the top 20 significantly enriched KEGG pathways (false discovery rate < 0.05). The results indicated that these targets are mainly related to the following signaling pathways, such as AGE-RAGE signaling pathway in diabetic complications, Coronavirus disease-COVID-19, IL-17 signaling pathway, Th17 cell differentiation and Toll-like receptor signaling pathway.

### 3.6. C-T-P network construction and analysis

**Figure 6.**
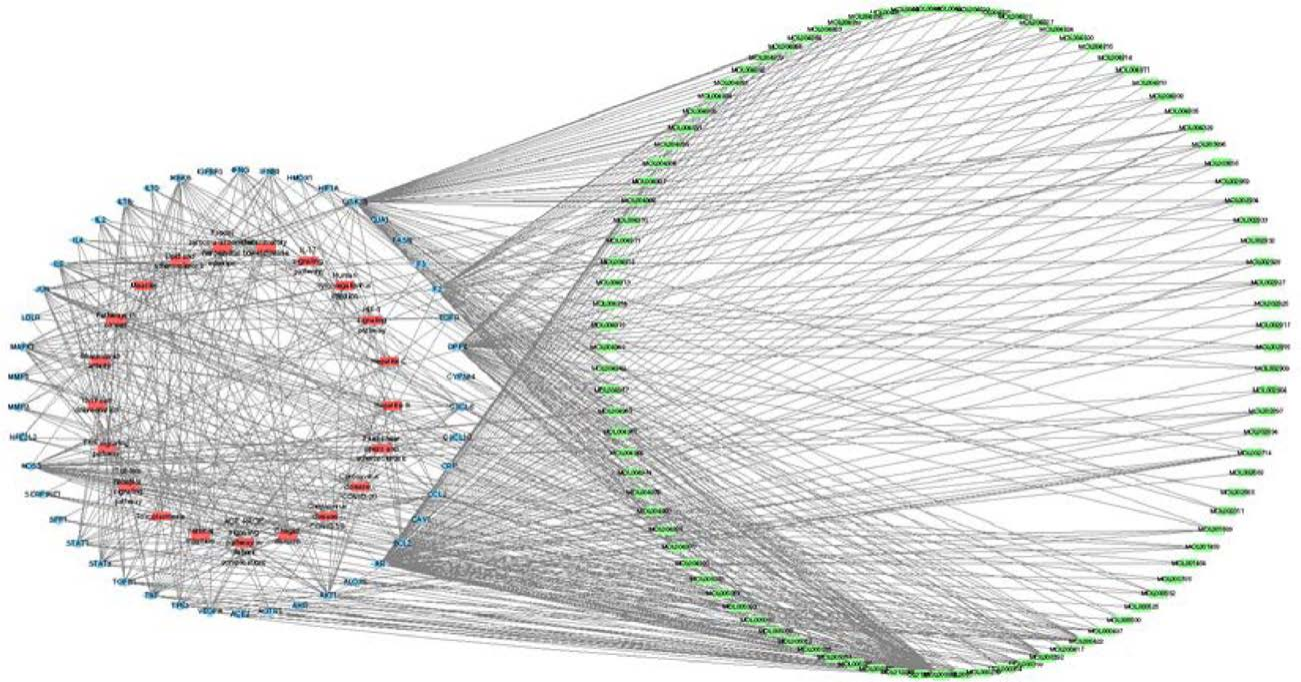
C-T-P for GGQL as a treatment for COVID-19 combined with DM network construction. Core pathways obtained by analyzing C-T-P networks (green nodes denote compounds, red represents pathways, and blue represents targets). Through C-T-P network analysis, quercetin (MOL000098, degree value = 75), kaempferol (MOL000422, degree value = 15) and formononetin (MOL000392, degree value = 14) have high degree value. It is suggested that it may be the key active ingredient in anti-COVID-19 patients with diabetes. The top 5 targets were AR (degree = 93), GSK3B (degree = 70), DPP4 (degree = 52), F2 (degree = 43) and NOS3 (degree = 34), and their candidate targets may be the main targets of GGQL against COVID-19 combined with diabetes mellitus. The 5 routes selected include Coronavirus disease-COVID-19 (degree = 18), AGE-RAGE signaling pathway in diabetic complications (degree = 17), and IL-17 signaling pathway (degree = 14), human cytomegalovirus infection (degree = 14) and Th17 cell differentiation (degree = 13). AR = androgens receptors, C-T-P = compound-target-pathway, DM = diabetes mellitus, GGQL = Gegen Qinlian decoction.

In order to construct the “C-T-P” network as shown in Figure 6, C-T-P network analysis revealed quercetin (MOL000098, degree = 75), kaempferol (MOL000422, degree = 15), and formononetin (MOL000392, degree = 14) possess high degree value, suggesting that it may be a key active ingredient in the fight against COVID-19 comorbid with DM.

Through the analysis of C-T-P network, 5 core targets of GGQL in the treatment of COVID-19 comorbid with DM and 5 important signaling pathways were screened out. The top 5 targets were AR (degree = 93), GSK3B (degree = 70), DPP4 (degree = 52), F2 (degree = 43) and NOS3 (degree = 34), and their candidate targets may be the main targets of GGQL against COVID-19 comorbid with DM. The 5 pathways include Coronavirus disease-COVID-19(degree = 18), AGE-RAGE signaling pathway in diabetic complications (degree = 17), IL-17 signaling pathway (degree = 14), Human cytomegalovirus infection (degree = 14) and Th17 cell differentiation(degree = 13) (see Table 3 for details).

**Table 3:** Based on the KEGG enrichment and C-T-P network analysis, we picked out 5 important signaling pathways that were significantly associated with GGQL treatment of COVID-19 complicated with DM.

Term	ID	Input number	*P* Value	Input gene name
Coronavirus disease – COVID-19	hsa05171	19	3.57E-17	JUN, CXCL8, IFNB1, STAT1, MMP1, STAT3, MMP3, F2, TNF, EGFR, IL2, IKBKB, ACE2, CXCL10, IL6, IL1B, AGTR1, CCL2, MAPK1
AGE-RAGE signaling pathway in diabetic complications	hsa04933	17	2.11E-20	JUN, TGFB1, CXCL8, NOS3, STAT1, STAT3, SERPINE1, F3, TNF, VEGFA, IL6, IL1B, BCL2, AGTR1, CCL2, AKT1, MAPK1
IL-17 signaling pathway	hsa04657	14	9.45E-16	GSK3B, JUN, CXCL8, MMP1, MMP3, TNF, IL4, IKBKB, CXCL10, IL6, IFNG, IL1B, CCL2, MAPK1
Human cytomegalovirus infection	hsa05163	14	8.59E-11	GSK3B, CXCL8, IFNB1, STAT3, TNF, EGFR, VEGFA, IKBKB, IL6, IL1B, CCL2, AKT1, MAPK1, TP53
Th17 cell differentiation	hsa04659	13	2.15E-13	JUN, TGFB1, STAT1, STAT3, AHR, HIF1A, IL2, IL4, IKBKB, IL6, IFNG, IL1B, MAPK1

C-T-P = compounds-target-pathway, DM = diabetes mellitus, GGQL = Gegen Qinlian decoction, KEGG = Kyoto Encyclopedia of Genes and Genomes.

### 3.7. Molecular docking study

**Figure 7.**
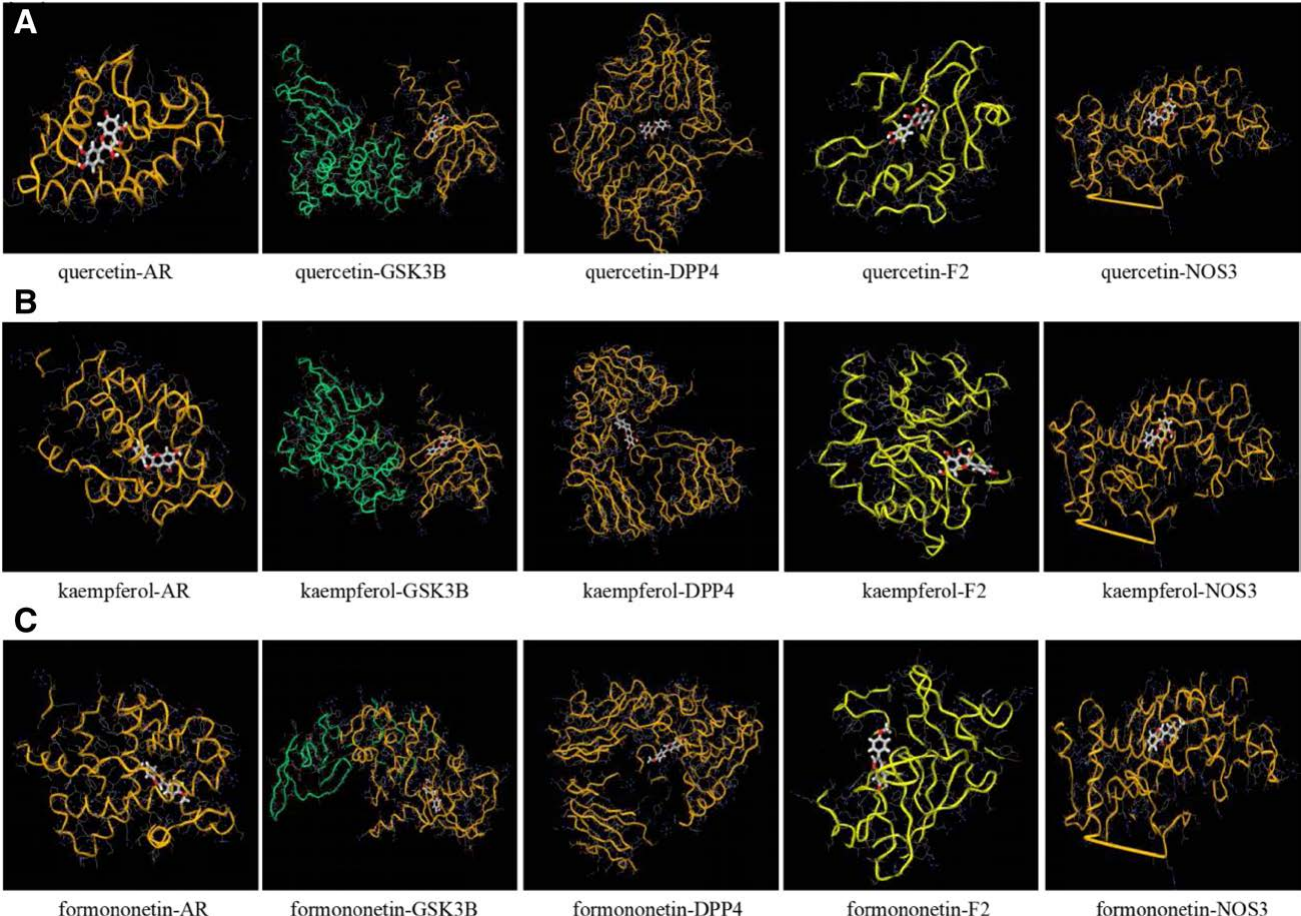
Docking pattern diagram of active components and key target molecules in GGQL. (A) quercetin interacts with key targets AR, GSK3B, DPP4, F2 and NOS3, respectively, (B) kaempferol interacts with key targets AR, GSK3B, DPP4, F2 and NOS3, respectively, (C) formononetin interacts with key targets AR, GSK3B, DPP4, F2 and NOS3, respectively. AR = androgens receptors, GGQL = Gegen Qinlian decoction.

By combining “PPI” and “C-T-P” network analysis, GGQL core active ingredients were quercetin, kaempferol and formononetin, and the binding ability was predicted with key targets AR, GSK3B, DPP4, F2 and NOS3. It is generally believed that binding energy less than −4.25 kcal·mol^−1^ indicates certain binding activity between ligand and receptor, less than −5.0 kcal·mol^−1^ indicates good binding activity, and less than −7.0 kcal·mol^−1^ indicates strong binding activity. Through docking of 3 active components with 5 target proteins using the mcule docking platform (https://mcule.com), it is found that quercetin, kaempferol and formononetin have good binding abilities with AR, GSK3B, DPP4, F2 and NOS3 (see Table 4; Fig. 7).

**Table 4:** Prediction of binding energy between active ingredients and key targets in the GGQL.

Compounds	AR	GSK3B	DPP4	F2	NOS3
quercetin	−8.7	−8.2	−7.6	−8.4	−9.1
kaempferol	−8.4	−8.0	−7.2	−8.2	−9.5
formononetin	−8.7	−7.9	−7.2	−8.0	−9.0

AR = androgens receptors, GGQL = Gegen Qinlian decoction.

## 4. Discussion

Diabetic patients are at higher risk of complications when infected with COVID-19. Poor prognosis is closely related to poor blood glucose control, and COVID-19 infection can cause blood glucose fluctuation and induce diabetic ketoacidosis, even to the extent of being lethal. Therefore, it is of great significance that effective intervention methods be developed for COVID-19 comorbid with DM. The TCM GGQL has been reported to have pharmacological effects in the treatment of type 2 diabetes and has played an important role in the clinical treatment of COVID-19. By conducting network pharmacology and molecular docking, this study explored the potential active ingredients and mechanism of action of GGQL in the treatment of COVID-19 comorbid with DM, aiming to provide a theoretical basis for the development of GGQL as adjuvant therapy for those affected by both diseases.

In this study, 127 main active components and 283 corresponding targets of GGQL were obtained, among which, 46 targets were found to co-act with COVID-19 comorbid with DM. Network analysis showed that the main active components of GGQL (quercetin, kaempferol, formononetin, etc.) may regulate AR, GSK3B, DPP4, F2, NOS3, and other targets. The results also indicate that these components act on various signaling pathways, such as the AGE-RAGE signaling pathway implicated in diabetic complications, the IL-17 signaling pathway, pathways related to human cytomegalovirus infection, and Th17 cell differentiation. These BPs may therefore mediate the way in which GGQL treats COVID-19 comorbid with DM.

Modern pharmacological studies have shown that quercetin, a component of GGQL, exhibits anti-COVID-19 activity by inhibiting the expression of human ACE2 receptors and several enzymes of SARS-CoV-2 (MPro, PLPro, and RdRp)^[12]^; meanwhile, Quercetin also exerts beneficial effects on T2DM, potentially by inhibiting pancreatic iron deposition and PBC ferroptosis.^[13]^ In vivo findings have also demonstrated it to be a promising agent against diabetes and its pathophysiological complications.^[14]^ Another component of GGQL, kaempferol, has excellent anti-diabetic effects; studies have shown that it can help to regulate lipid metabolism, improve IR, reduce lipotoxicity, improve insulin signaling, and restore the balance between glucose utilization and production to play an anti-diabetic role^[15]^; Kaempferol is also considered a candidate compound for the treatment of COVID-19.^[16]^ Its anti-COVID-19 effects have been associated with the regulation of inflammation, oxidative stress, immunity, virus infection, cell growth, and metabolism.^[17]^ Also contained within GGQL, formononetin is a flavonoid that may be a promising inhibitor of SARS-CoV-2^[18]^ and has demonstrated its potential as a treatment against the infection.^[19]^ Formononetin treatment has been found to reduce insulin resistance and protect against pancreatic β-cell apoptosis caused by IL-1β to attenuate hyperglycemia in type 2 diabetes.^[20,21]^ These studies demonstrate the efficacy of GGQL’s active components in the treatment of COVID-19 comorbid with DM.

Through network analysis, it was found that the core targets of GGQL in the treatment of COVID-19 comorbid with DM include AR, GSK3B, DPP4, F2, NOS3, and so on. Androgens are believed to play an important role in the pathogenesis of COVID-19, as recent studies and international statistics have shown increased prevalence, morbidity, and mortality rates of COVID-19 in male patients compared to female patients.^[22]^ The regulation of androgens and their receptors (AR) are important factors modulating the severity of COVID-19.^[23]^ Moreover, the expression of AR plays a role in the development of type 2 diabetes and insulin resistance.^[24]^ The phosphorylation of the N protein produced by SARS-CoV-2 by glycogen synthase kinase 3 (GSK3) is required for its function, while the inhibition of GSK3B impairs its phosphorylation, viral transcription, and replication.^[25]^ DPP4/CD26 is a single-pass transmembrane protein with multiple functions, including glycemic control, cell migration, cell proliferation, and immunity, among others. *In silico* experiments have suggested that SARS-CoV-2 might bind DPP4/CD26 to mediate its infection.^[26]^ In particular, the distribution of DPP4 in the human respiratory tract may facilitate the entrance of the virus into the airway tract itself, contributing to the development of a cytokine storm and immunopathology, and causing fatal COVID-19 pneumonia.^[27]^ DPP4 is also an important target in the treatment of diabetes and is a relevant factor linking the risk of SARS-CoV-2 infection and the severity of COVID-19 in diabetic patients.^[28]^ Thrombin (F2) is a trypsin-like serine protease with multiple physiological functions. Depending on the pathogenesis of COVID-19, F2 inhibitors may confer a variety of potential therapeutic benefits, including anti-thrombotic, anti-inflammatory, and anti-viral activities.^[29,30]^ Noteworthily, 80% of diabetic patients die of thrombotic death, so F2 is also an important target for the prevention and treatment of diabetes.^[31]^ Studies have found that polymorphisms in the *NOS3* gene are closely related to susceptibility to COVID-19^[32]^ and susceptibility to vascular disease in patients with type 2 diabetes,^[33]^ indicating this gene to be an important target for the treatment of both diseases.

Herein, molecular docking technology was used to predict the binding ability of GGQL’s main active ingredients (quercetin, kaempferol, formononetin, etc.) and core targets (AR, GSK3B, DPP4, F2, and NOS3). It was found that quercetin, kaempferol and formononetin had strong binding ability with AR, GSK3B, DPP4, F2, and NOS3 (binding energy all less than −7.0 kcal·mol^−1^ that indicates strong binding activity), respectively, and the docking of kaempferol and NOS3 showed the lowest binding energy, suggesting that the combination was the most stable. As several reports found that the quercetin and kaempferol play a role in the treatment of coronavirus infection by regulating the body’s immunity, fighting inflammation, and antiviral activities by acting on multiple targets (such as NOS3, GSK3B, etc.)^[34]^ and also plays an important role to prevent and/or ameliorate T2DM-related complications.^[35]^ These results suggest that these active components and their corresponding targets are largely responsible for the therapeutic effect of GGQL against COVID-19 comorbid with DM.

In summary, this study adopted the methods of network pharmacology and molecular docking to explore the mechanism of GGQL in the treatment of COVID-19 comorbid with DM. This approach revealed the effect of GGQL on COVID-19 comorbid with DM from the aspects of multiple components, multiple targets, and multiple pathways, providing a basis for clinical treatment. However, this study has shortcomings. For example, the collection of bioactive ingredients and their potential targets from databases was not comprehensive, while the key targets and pathways obtained from network pharmacology still need to be further verified both by in vitro and in vivo experiments. Nevertheless, the findings indicate that the traditional Chinese prescription GGQL may have good clinical value in the treatment of COVID-19 comorbid with diabetes. Therefore, we hope that these findings may lead to future pharmacology-based research on other Chinese herbal medicines to provide additional therapeutic means for the treatment of COVID-19 comorbid with DM and other related diseases.

## Acknowledgments

Firstly, I would like to express my gratitude to Dr Zhi-Qiang Li and Dr Shan-shan Lei for his guidance on experimental design. Secondly, I would like to express my gratitude to Cong Zhou and Pei Wang for their contributions in data collection. I would also like to express my gratitude to Qing-hua Ke and Jie Zhang for their timely assistance in data statistical analysis. Finally, we would like to express our gratitude to the Public Health Project of Hubei Province (WJ2023M173) and the Science and Technology Project of Jingmen City (2020YFYB075 and 2021YFYB062) for their financial support for this study.

## Author contributions

**Conceptualization:** Zhi-qiang Li.

**Data curation:** Cong Zhou, Lin-zi Li, Pei Wang.

**Formal analysis:** Qing-hua Ke, Jie Zhang.

**Funding acquisition:** Lin-zi Li.

**Project administration:** Shan-shan Lei.

**Writing – original draft:** Cong Zhou, Lin-zi Li.

**Writing – review & editing:** Zhi-qiang Li, Shan-shan Lei.

## Supplementary Material
